# First-principles calculations of perpendicular magnetic anisotropy in Fe_1−*x*_Co_*x*_/MgO(001) thin films

**DOI:** 10.1186/s11671-015-0825-3

**Published:** 2015-03-12

**Authors:** Guanzhi Cai, Zhiming Wu, Fei Guo, Yaping Wu, Heng Li, Qianwen Liu, Mingming Fu, Ting Chen, Junyong Kang

**Affiliations:** Department of Physics, Fujian Key Laboratory of Semiconductor Materials and Applications, Xiamen University, 422 Siming South Road, Xiamen, 361005 People’s Republic of China

**Keywords:** First-principles calculations, FeCo/MgO, Perpendicular magnetic anisotropy

## Abstract

The perpendicular magnetic anisotropy (PMA) of Fe_1−*x*_Co_*x*_ thin films on MgO(001) was investigated via first-principles density-functional calculations. Four different configurations were considered based on their ground states: Fe/MgO, Fe_12_Co_4_/MgO, Fe_10_Co_6_/MgO, and Fe_8_Co_8_/MgO. As the Co composition increases, the amplitude of PMA increases first from Fe/MgO to Fe_12_Co_4_/MgO, and then decreases in Fe_10_Co_6_/MgO; finally, the magnetic anisotropy becomes horizontal in Fe_8_Co_8_/MgO. Analysis based on the second-order perturbation of the spin-orbit interaction was carried out to illustrate the contributions from Fe and Co atoms to PMA, and the differential charge density was calculated to give an intuitive comparison of 3*d* orbital occupancy. The enhanced PMA in Fe_12_Co_4_/MgO is ascribed to the optimized combination of occupied and unoccupied 3*d* states around the Fermi energy from both interface Fe and Co atoms, while the weaker PMA in Fe_10_Co_6_/MgO is mainly attributed to the modulation of the interface Co-*d*_*xy*_ orbital around the Fermi energy. By adjusting the Co composition in Fe_1−*x*_Co_*x*_, the density of states of transitional metal atoms will be modulated to optimize PMA for future high-density memory application.

## Background

Materials with large magnetic moments and strong perpendicular magnetic anisotropy (PMA) are of great interest due to their potential applications in next-generation high-density non-volatile memories and high thermal stability logic chips [1-6]. Numbers of materials with strong PMA have been explored during the past decades, such as L1_0_-ordered (Co,Fe)-Pt alloys [7-9], Co/(Pd,Pt) multilayers [10-12], and *D*0_22_-ordered Mn_3−δ_Ga [13,14]. However, none of them satisfy the high thermal stability, low switching current, and high tunnel magnetoresistance (TMR) ratio at the same time. Recently, FeCo alloys with high saturation magnetization, high Curie temperature, good permeability, and large magnetocrystalline anisotropy energy have been paid great attention [15]. The large values of uniaxial magnetocrystalline anisotropy energy (*K*_u_) and saturation magnetization (*M*_s_) were first predicted by Burkert et al. via first-principles calculations [16] and then verified by experiments [17,18]. Particularly, S. Ikeda et al. obtained Ta/FeCoB/MgO/FeCoB/Ta perpendicular magnetic tunnel junctions with high TMR ratio (over 120%), high thermal stability at a dimension of 40 nm diameter, and a low switching current of 49 μA [19], revealing a promising building block for future high-density memories. After that, lots of experiments based on Fe_1−*x*_Co_*x*_/MgO magnetic tunnel junctions have been performed to explore the influence of growth regulation, electric field, Fe-Co proportion, and so on [20-23]. Though it has been proved by experiments that Fe-rich Fe_1−*x*_Co_*x*_B/MgO structures have larger PMA than their Co-rich counterparts, there is short of theoretical guidance for optimizing Fe-Co proportion, and the inherent origin of PMA in Fe_1−*x*_Co_*x*_B/MgO is still unclear.

In this work, we investigated the amplitude of PMA depending on the cobalt composition and explored the origin of PMA in Fe_1−*x*_Co_*x*_/MgO magnetic tunnel junctions via first-principles calculations. Second-order perturbation theory was adopted to illustrate the contributions from Fe and Co atoms to PMA. The differential charge density at the sites where Co atoms take the place of Fe atoms was calculated to give an intuitive comparison of 3*d* orbital occupancy. By adjusting the cobalt composition, the density of states (DOS) will be modulated, and strong PMA will be obtained with an optimized combination of occupied and unoccupied 3*d* states around the Fermi energy (*E*_F_).

## Methods

**Figure 1:**
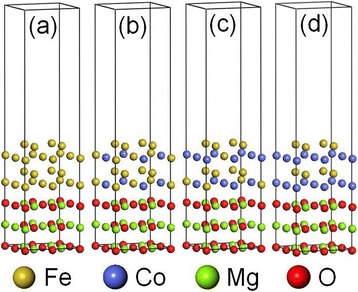
**Schematics of the calculated structures. (a)** Fe/MgO, **(b)** Fe_12_Co_4_/MgO, **(c)** Fe_10_Co_6_/MgO, and **(d)** Fe_8_Co_8_/MgO. Fe, Co, Mg, and O atoms are represented by yellow, blue, green, and red balls, respectively.

We performed first-principles density-functional calculations using the Vienna *ab initio* simulation package (VASP) with the consideration of the spin-orbit interactions. For the electronic exchange-correlation and electron-ion interaction, we adopted the spin-polarized generalized gradient approximation (GGA) [24] and the projector-augmented wave (PAW) potential [25], respectively. A 9 × 9 × 1 *k*-point mesh was used with the energy cutoff equal to 500 eV. Four 2 × 2 supercells with different Co concentrations were considered in this work: Fe/MgO(001), Fe_12_Co_4_/MgO(001), Fe_10_Co_6_/MgO(001), and Fe_8_Co_8_/MgO(001) (Figure 1). The configurations of FeCo alloys were given as their ground states according to previous work, i.e., L6_0_-Fe_12_Co_4_, Fe_10_Co_6_, and B2-Fe_8_Co_8_ (CsCl type) [26]. Along the *z*-axis, there were three MgO monolayers, four Fe_1−*x*_Co_*x*_ monolayers and 15 Å vacuum. Each supercell includes 40 atoms. The bottom MgO monolayer was fixed as bulk, and the in-plane lattice constant of the supercell was fixed at $\sqrt{2}\mathrm{a}$ (cubic MgO: *a* = 4.212 Å) since a thin ferromagnetic layer was used. All the other layers were fully relaxed until the largest force between the atoms worked out to be less than 1 meV/Å. The magnetic anisotropy energy (MAE) was calculated by taking the difference between the total energy of the magnetization oriented along the in-plane [100] and out-of-plane [001] directions based on the force theorem.

## Results and discussion

The calculated MAE in Fe_1−*x*_Co_*x*_/MgO (*x* = 0, 0.25, 0.375, 0.5) configurations are listed in Table 1. Fe/MgO, Fe_12_Co_4_/MgO, and Fe_10_Co_6_/MgO have a positive MAE of 4.2, 7.1, and 3.3 meV, respectively, indicating the preference of out-of-plane magnetization. However, Fe_8_Co_8_/MgO has a negative MAE of −17.1 meV, showing an in-plane easy magnetization. In Fe_8_Co_8_/MgO, the interfacial Fe atoms are replaced by Co atoms, and the calculated magnetic moment of O atoms in Co-O bonds is 0.013 μ_B_, only half of that in Fe-O bonds (0.025 μ_B_), indicating the reduced transition metal-oxygen hybridization. As revealed by Yang et al., the hybridization between Fe and O atoms at interface greatly contributed to PMA in Fe/MgO [27]. Thus, the attenuated interface hybridization in Fe_8_Co_8_/MgO may be an origin of the in-plane easy magnetization. Furthermore, for FeCo monolayers epitaxially grown on MgO (001), the in-plane lattice is enlarged to match that of MgO(001) (*a* = 2.97 Å), while the out-of-plane lattice is shortened to 2.71 Å, leading to a *c*/*a* ratio of 0.91. It has been reported that the MAE reduces as the *c*/*a* ratio decreases and becomes negative when it is smaller than 1 for B2-FeCo alloy [28]. Hence, a negative MAE is anticipated in Fe_8_Co_8_/MgO. In a word, the replacement of interface Fe atoms with Co atoms and the small *c*/*a* ratio in Fe_8_Co_8_/MgO accounts for the in-plane magnetization.

**Table 1:** **MAE values and interfacial distances of Fe-O and Co-O bonds in Fe**_1−x_ **Co**_x_ **/MgO systems**

	**Distance (Fe-O Ǻ)**	**Distance (Co-O Ǻ)**	**MAE (meV)**
Fe/MgO	2.136	\	4.2
Fe_12_CO_4_/MgO	2.073	2.164	7.1
Fe_10_CO_6_/MgO	2.079	2.127	3.3
Fe_8_CO_8_/MgO	\	2.103	−17.1

**Figure 2:**
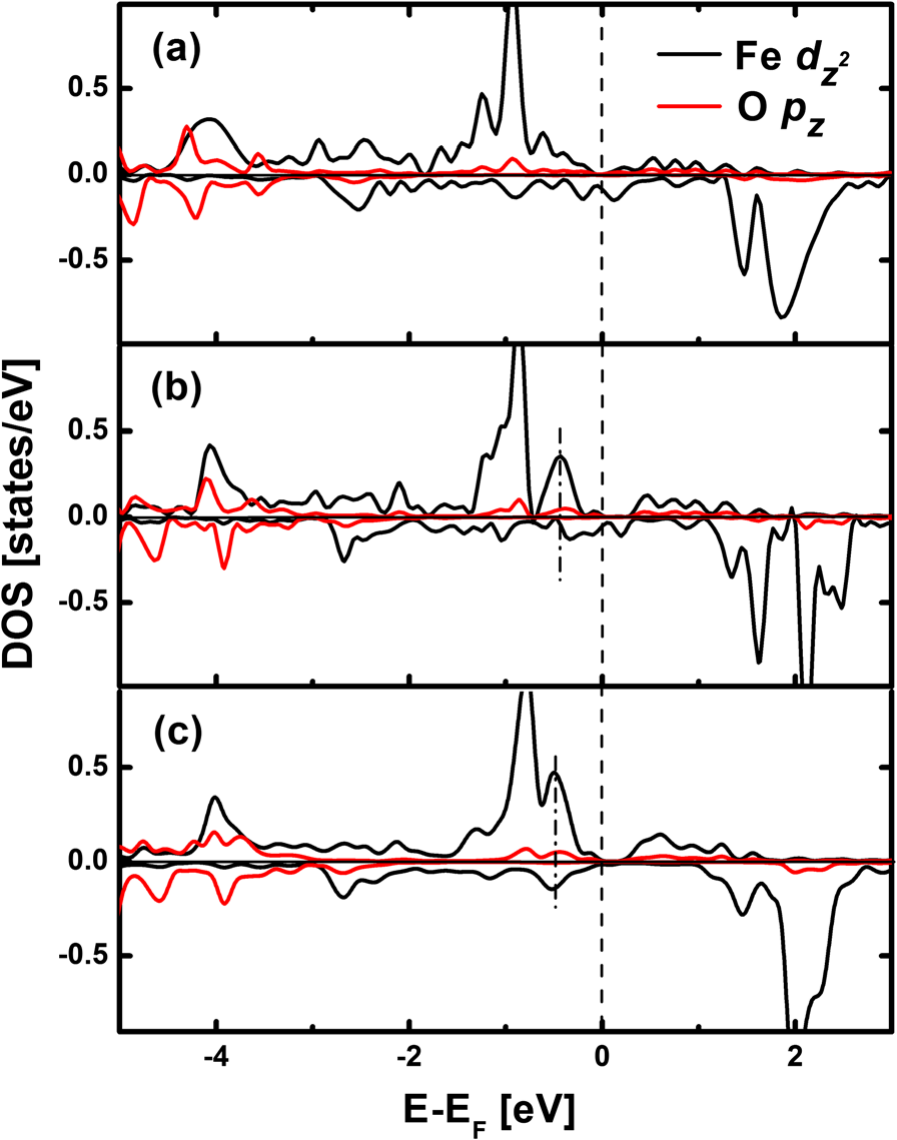
**Majority-spin (positive) and minority-spin (negative) DOS of** $\mathbf{F}\mathbf{e}\hbox{-} {\boldsymbol{d}}_{{\mathbf{z}}^{\mathbf{2}}}$ **and O-** ***p***_z_ **. (a)** Fe/MgO interface, **(b)** Fe_12_Co_4_/MgO interface, and **(c)** Fe_10_Co_6_/MgO interface as a function of energy relative to the *E*F.

Note that Fe_12_Co_4_/MgO has the largest PMA value, implying that a proper Fe-Co combination will enhance PMA. It has been reported that the PMA is dominated by the interfacial anisotropy in Fe_1−*x*_Co_*x*_/MgO systems [19]. Furthermore, the Fe-O hybridization makes the primary contribution to PMA in Fe/MgO systems. As shown in Table 1, the Fe-O distances in Fe/MgO, Fe_12_Co_4_/MgO, and Fe_10_Co_6_/MgO are 2.136, 2.073, and 2.079 Å, respectively. The change of the Fe-O distance will cause the reconstruction of the electronic structure of $\mathrm{F}\mathrm{e}\hbox{-} {d}_{z^2}$ around the *E*_F_. Figure 2 shows the DOS of $\mathrm{F}\mathrm{e}\hbox{-} {d}_{z^2}$ and O-*p*_*z*_ orbitals at Fe/MgO, Fe_12_Co_4_/MgO, and Fe_10_Co_6_/MgO interfaces. In the vicinity of *E*_F_, there are enhanced hybridization peaks indicated by dashed lines in Fe_12_CO_4_/MgO and Fe_10_Co_6_/MgO, implying an increased occupancy of $\mathrm{F}\mathrm{e}\hbox{-} {d}_{z^2}$ states compared with Fe/MgO. Meanwhile, Fe-*d*_*xz,yz*_ states hybridize with ${d}_{z^2}$ states through spin-orbital interaction, leading to enhanced *d*_*xz,yz*_ occupancy. According to the previous reports, *d*_*xz,yz*_ orbital plays an important role in the out-of-plane components of PMA [27]. Therefore, the Fe atoms in Fe_12_Co_4_/MgO and Fe_10_Co_6_/MgO will somehow increase the positive contribution to PMA due to the enhanced Fe-O hybridization.

**Figure 3:**
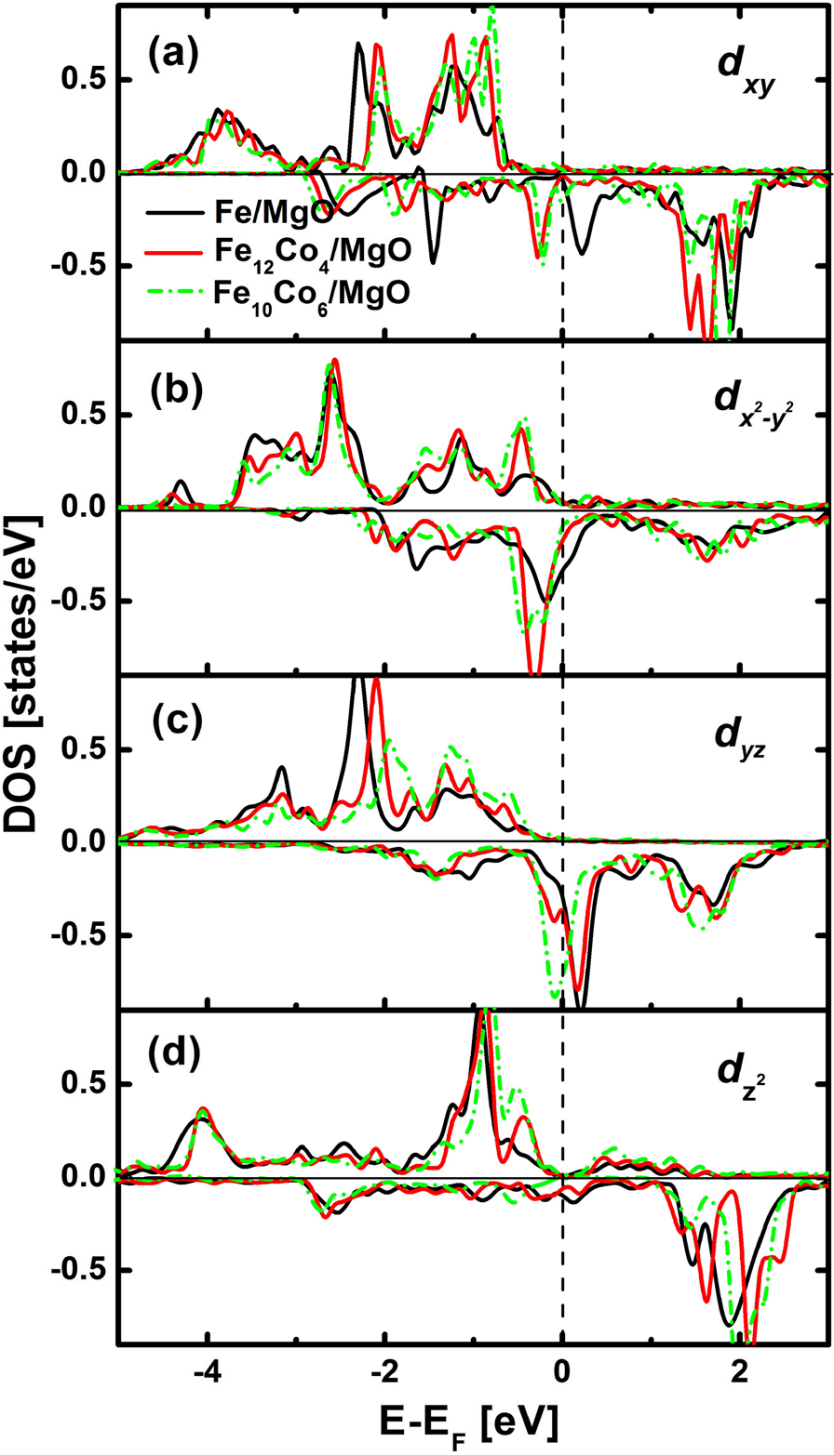
**Majority-spin (positive) and minority-spin (negative) DOS projected to Fe-3** ***d*** **orbital components (a-d).** Fe/MgO interface (black), Fe_12_Co_4_/MgO interface (red), and Fe_10_Co_6_/MgO interface (green).

To further understand the contributions from Fe atoms to PMA in Fe_1−*x*_Co_*x*_/MgO systems, we consider the second-order perturbative contribution of spin-orbit coupling (SOC) to magnetocrystalline anisotropy energies $\left({E}_{\mathrm{MCA}}^{(2)}\right)$ depending on the atomic site and the spin-transition process. ${E}_{\mathrm{MCA}}^{(2)}$ can be expressed by four terms (up-up, down-down, up-down, down-up) according to the Ref. [29]:

1 $$ \begin{array}{c}\hfill {E}_{\mathrm{MCA}}^{(2)}={\displaystyle \sum_i}{E}_{\mathrm{MCA}}^i\hfill \\ {}\hfill \kern5em ={\displaystyle \sum_i}\varDelta {E}_{\uparrow \Rightarrow \uparrow}^i+\varDelta {E}_{\downarrow \Rightarrow \downarrow}^i-\varDelta {E}_{\uparrow \Rightarrow \downarrow}^i-\varDelta {E}_{\downarrow \Rightarrow \uparrow}^i\hfill \end{array} $$

and

2 $$ \begin{array}{c}\hfill \varDelta {E}_{\sigma \Rightarrow {\sigma}^{\hbox{'}}}^i=-{\xi}_i{\displaystyle \sum_{\mu \lambda {\mu}^{\hbox{'}}{\lambda}^{\hbox{'}}}}\Big[\left.\lambda \uparrow \right|{L}_X\left|{\lambda}^{\hbox{'}}\uparrow \right.\left.{\mu}^{\hbox{'}}\uparrow \right|{L}_X\left|\mu \uparrow \right.\hfill \\ {}\hfill -\left.\lambda \uparrow \right|{L}_Z\left|{\lambda}^{\hbox{'}}\uparrow \right.\left.{\mu}^{\hbox{'}}\uparrow \right|{L}_Z\left|\mu \uparrow \right.\Big]\hfill \\ {}\hfill \times {\displaystyle \sum_j}{\xi}_j{G}_{\mu \lambda}^{\mu^{\hbox{'}}{\lambda}^{\hbox{'}}}\left(\sigma, {\sigma}^{\hbox{'}};i,j\right)\hfill \end{array} $$

where $\varDelta {E}_{\uparrow \Rightarrow \uparrow}^i$ (up-up) in Equation 1 indicates a virtual excitation from occupied majority-spin states to unoccupied majority-spin states of an atom in the second-order perturbation. In Equation 2, *ξ* is the SOC constant, *σ* is the spin state, *λ*(*μ*) is the atomic orbital state, the superscript *ʹ* represents the unoccupied state, and ${G}_{\mu \lambda}^{\mu^{\hbox{'}}{\lambda}^{\hbox{'}}}\left(\sigma, {\sigma}^{\hbox{'}};i,j\right)$ is an integral of the joint density of states given by

3 $$ \begin{array}{c}\hfill {G}_{\mu \lambda}^{\mu^{\hbox{'}}{\lambda}^{\hbox{'}}}\left(\sigma, {\sigma}^{\hbox{'}};i,j\right)={\displaystyle \underset{-\infty }{\overset{E_F}{\int }}}d\varepsilon {\displaystyle \underset{-\infty }{\overset{E_F}{\int }}}d{\varepsilon}^{\hbox{'}}\frac{1}{\varepsilon^{\hbox{'}}-\varepsilon}\hfill \\ {}\hfill \kern9.5em \times {\displaystyle \sum_k}{\displaystyle \sum_n^{\mathrm{occ}}}{c}_{i\mu \sigma}^{kn*}{c}_{j\lambda \sigma}^{kn}\delta \left(\varepsilon -{\varepsilon}_{kn\sigma}^{(0)}\right)\hfill \\ {}\hfill \kern9.75em \times {\displaystyle \sum_{n^{\hbox{'}}}^{\mathrm{unocc}}}{c}_{i{\mu}^{\hbox{'}}{\sigma}^{\hbox{'}}}^{k{n}^{\hbox{'}}*}{c}_{j{\lambda}^{\hbox{'}}{\sigma}^{\hbox{'}}}^{k{n}^{\hbox{'}}}\delta \left({\varepsilon}^{\hbox{'}}-{\varepsilon}_{k{n}^{\hbox{'}}{\sigma}^{\hbox{'}}}^{(0)}\right)\hfill \end{array} $$

where ${c}_{i\mu \sigma}^{kn}$ is the components of an orthogonal basis of atomic orbitals and ${\varepsilon}_{kn\sigma}^{(0)}$ is the energy of unperturbed state. It can be seen from Equations 2 and 3 that $\varDelta {E}_{\sigma \Rightarrow {\sigma}^{\hbox{'}}}^i$ depends not only on the coupling between the occupied states and unoccupied states but also strongly on the splitting between them through the energy denominator in Equation 3. As a result, $\varDelta {E}_{\sigma \Rightarrow {\sigma}^{\hbox{'}}}^i$ is mainly determined by the DOS in the vicinity of *E*_F_. Figures 3a,b,c,d show the DOS of Fe *d*_*xy*_, *d*_*xz,yz*_, ${d}_{z^2}$, and ${d}_{x^2-{y}^2}$ at Fe/MgO, Fe_12_CO_4_/MgO, and Fe_10_Co_6_/MgO interfaces as a function of energy relative to *E*_F_. The majority-spin states are almost fully occupied, and the minority-spin states are partially occupied for Fe *d*_*xy*_, *d*_*xz,yz*_, ${d}_{z^2}$, and ${d}_{x^2-{y}^2}$ orbitals. According to Equations 2 and 3, $\varDelta {E}_{\downarrow \Rightarrow \uparrow}^{\mathrm{Fe}}$ (i.e., the coupling between occupied minority-spin states and unoccupied majority-spin states) and $\varDelta {E}_{\uparrow \Rightarrow \uparrow}^{\mathrm{Fe}}$ (i.e., the coupling between occupied majority-spin states and unoccupied majority-spin states) can be neglected. The spin-flip term $\varDelta {E}_{\uparrow \Rightarrow \downarrow}^{\mathrm{Fe}}$ and the spin-conservation term $\varDelta {E}_{\downarrow \Rightarrow \downarrow}^{\mathrm{Fe}}$ make the main contributions to the MAE. To estimate $\varDelta {E}_{\sigma \Rightarrow {\sigma}^{\hbox{'}}}^i$, the angular momentum matrix elements are considered. Due to symmetry property of the atomic orbitals, only a few angular momentum matrix elements between the *d* orbitals are nonzero: 〈*xz*|*L*_*z*_|*yz*〉, 〈*x*^2^−*y*^2^|*L*_*z*_|*xy*〉, 〈*z*^2^|*L*_*x*_|*yz*〉, 〈*xy*|*L*_*x*_|*xz*〉, and 〈*x*^2^−*y*^2^|*L*_*x*_|*yz*〉 (bra and ket can be exchanged). Among which, 〈*μ*|*L*_*z*_|*μ*′〉 makes a positive contribution to PMA through spin-conservation term $\varDelta {E}_{\downarrow \Rightarrow \downarrow}^i$ and a negative contribution through spin-flip term $\varDelta {E}_{\uparrow \Rightarrow \downarrow}^i$, opposite does 〈*μ*|*L*_*x*_|*μ*′〉. In Fe/MgO, the matrix element 〈*x*^2^−*y*^2^|*L*_*z*_|*xy*〉 makes the main positive contribution to PMA due to the large occupied ${d}_{x^2-{y}^2}$ and unoccupied *d*_*xy*_ states of minority-spin around *E*_F_. The primary negative contribution comes from 〈*x*^2^−*y*^2^|*L*_*x*_|*yz*〉 and 〈*yz*|*L*_*x*_|*x*^2^−*y*^2^〉 on account of large unoccupied *d*_*yz*_ states and relative large unoccupied ${d}_{x^2-{y}^2}$ states around *E*_F_. To sum up, the PMA value contributed from all nonvanishing angular momentum matrix elements connecting *d* states $\pm {\left|\left.{\mu}^{\hbox{'}}\right|L\left|\mu \right.\right|}^2{G}_{\mu \mu}^{\mu {\mu}^{\hbox{'}}}\left(\sigma, \downarrow; \mathrm{F}\mathrm{e},\mathrm{F}\mathrm{e}\right)$ is positive. Different from Fe/MgO, the matrix elements 〈*x*^2^−*y*^2^|*L*_*z*_|*xy*〉 in Fe_12_Co_4_/MgO and Fe_10_Co_6_/MgO are quite small because of the greatly reduced unoccupied minority-spin *d*_*xy*_ states. The other obvious changes are the increase of occupied minority-spin *d*_*yz*_ states in both Fe_12_Co_4_/MgO and Fe_10_Co_6_/MgO and the decrease of unoccupied minority-spin ${d}_{x^2-{y}^2}$ states, which will amplify the positive value of 〈*xz*|*L*_*z*_|*yz*〉 and diminish the negative value of 〈*yz*|*L*_*x*_|*x*^2^−*y*^2^〉. As a result, the contributions from Fe atoms in Fe_12_Co_4_/MgO and Fe_10_Co_6_/MgO to PMA are larger than those in Fe/MgO, which confirms the speculation based on Fe-O hybridization.

**Figure 4:**
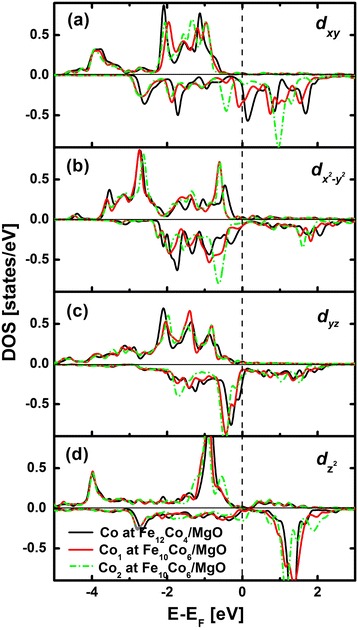
**Majority-spin (positive) and minority-spin (negative) DOS projected to Co-3** ***d*** **orbital components (a-d).** Fe_12_Co_4_/MgO interface (black) and Fe_10_Co_6_/MgO interface. Co_1_ (red) represents the Co atom at the edge of the interface, and Co_2_ (green) represents the Co atom in the center of the interface.

In the following, the contributions from Co atoms are taken into consideration. The DOS of Co-*d*_*xy*_, *d*_*xz,yz*_, ${d}_{z^2}$, and ${d}_{x^2-{y}^2}$ at Fe_12_Co_4_/MgO and Fe_10_Co_6_/MgO interfaces are shown in Figure 4a,b,c,d. Since Co atoms at Fe_10_Co_6_/MgO interface are not symmetrical, we present their DOS separately. Co_1_ represents the Co atom at the edge of the interface, and Co_2_ represents the Co atom in the center of the interface. Different from Fe-*d*_*yz*_ states, the unoccupied Co-*d*_*yz*_ states around *E*_F_ are relatively small in Fe_12_Co_4_/MgO; thus, the negative matrix element 〈*x*^2^−*y*^2^|*L*_*x*_|*yz*〉 is much smaller than that of Fe/MgO. The other negative contribution arising from 〈*yz*|*L*_*x*_|*xy*〉 is comparable to the negative matrix element 〈*yz*|*L*_*x*_|*x*^2^−*y*^2^〉 in Fe/MgO. In addition, the positive contribution of *L*_*z*_ connecting occupied ${d}_{x^2-{y}^2}$ and unoccupied *d*_*xy*_ states in Fe_12_Co_4_/MgO is comparable to that in Fe/MgO. In general, Co atoms in Fe_12_Co_4_/MgO contribute much more to PMA than Fe atoms in Fe/MgO. In the light of these, the PMA value contributed from both Fe and Co atoms is much larger in Fe_12_Co_4_/MgO than in Fe/MgO. In Fe_10_Co_6_/MgO, the positive matrix element 〈*x*^2^−*y*^2^|*L*_*z*_|*xy*〉 decreases due to the reduced unoccupied *d*_*xy*_ states for Co_1_ atom. It deceases even more for Co_2_ atom because of the very small unoccupied *d*_*xy*_ states. Consequently, the contribution from Co atoms in Fe_10_Co_6_/MgO is much smaller than Co atoms in Fe_12_Co_4_, resulting in a relative small value of PMA.

**Figure 5:**
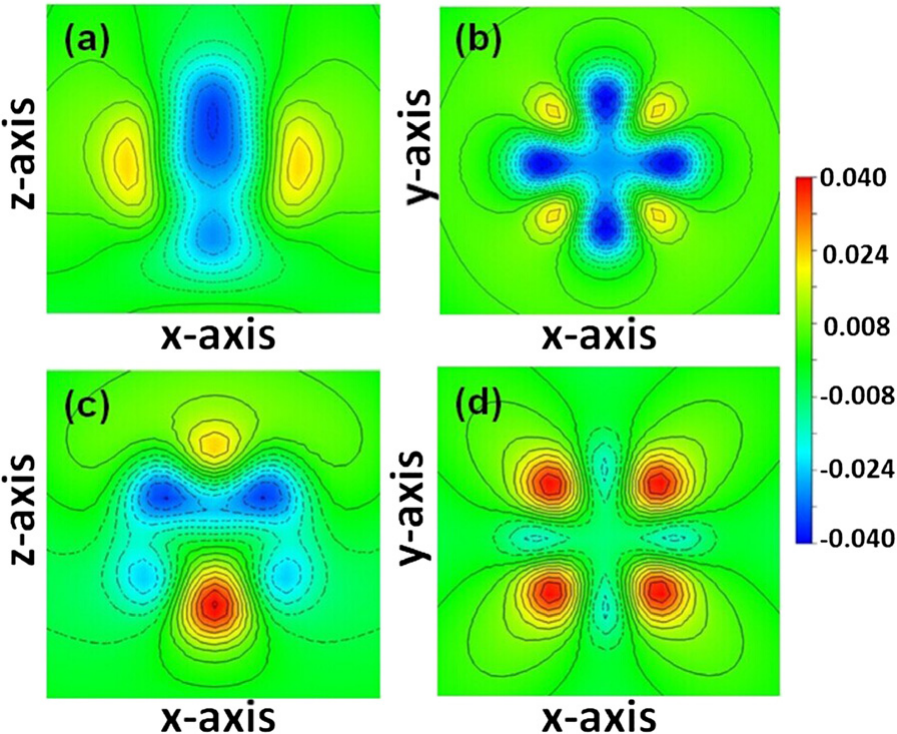
**The differential charge density. (a)**, **(b)** Fe/MgO and Fe_12_Co_4_/MgO interfaces where Co take Fe site; **(c)**, **(d)** Fe_12_Co_4_/MgO and Fe_10_Co_6_/MgO interfaces where extra Co take center Fe site.

In order to give a more intuitive comparison of the contributions of Fe and Co atoms, we calculated the differential charge density. Figures 5a,b shows the differential charge density of Fe_12_Co_4_/MgO and Fe/MgO at the sites where Fe atoms are replaced by Co atoms at interface. Compared with Fe atoms in Fe/MgO, Co atoms in Fe_12_Co_4_/MgO have larger *d*_*xz,yz*_ and *d*_*xy*_ occupancy but smaller ${d}_{x^2-{y}^2}$ and ${d}_{z^2}$ occupancy. The occupancy of ${d}_{z^2}$, *d*_*xz*_, and *d*_*yz*_ orbitals determine the atomic orbital magnetic moments of [001], while the occupancy of *d*_*xy*_ and ${d}_{x^2-{y}^2}$ orbitals contribute to [100] [30]. Due to the sharply reduced ${d}_{x^2-{y}^2}$ occupancy, Co atoms in Fe_12_Co_4_/MgO contribute more to PMA than Fe atoms in Fe/MgO, which is consistent with the estimation from the second-order perturbation of SOC. Figures 5c,d display the differential charge density of Fe_10_Co_6_/MgO and Fe_12_Co_4_/MgO at the center of the interface where Co atom replace Fe atom. The ${d}_{z^2}$ occupancy increases, but the *d*_*xz,yz*_ occupancy largely decreases. In addition, the *d*_*xy*_ states enhance dramatically. As a result, Co_2_ atom in Fe_10_Co_6_/MgO contributes much less to PMA than Fe atom in Fe_12_Co_4_/MgO, which agrees well with the estimation from the second-order perturbation of SOC.

## Conclusions

In summary, we have presented first-principles studies of PMA in Fe_1−*x*_Co_*x*_/MgO as a function of the Co composition and strong PMA with an amplitude of 7.1 mV is discovered in Fe_12_Co_4_/MgO. The change of Co composition modulates the DOS of Fe and Co atoms around *E*_F_ and varies their contributions to PMA. The enhanced PMA in Fe_12_Co_4_/MgO is ascribed to the optimized combination of interface occupied and unoccupied 3*d* states around *E*_F_, where both Fe and Co atoms make a large out-of-plane contribution and a small in-plane contribution. The weaker PMA in Fe_10_Co_6_/MgO is due to the sharply decreased unoccupied Co-*d*_*xy*_ states around *E*_F_ and dramatically increased Co-*d*_*xy*_ occupancy, where Co atoms especially the Co atom at the center of interface make a small out-of-plane contribution. The horizontal magnetic anisotropy in Fe_8_Co_8_/MgO is mainly resulted from the attenuation of interface hybridization and the small *c*/*a* ratio. Therefore, reasonable adjustment of Fe-Co combination in Fe_1−*x*_Co_*x*_/MgO magnetic tunnel junctions will enhance PMA and is worthy of further investigation. This work provides a basis method to investigate promising building blocks for future high-density memories.
